# The Dual/Global Value of SARS-CoV-2 Genome Surveillance on Migrants Arriving to Europe via the Mediterranean Routes

**DOI:** 10.5334/aogh.3425

**Published:** 2021-07-21

**Authors:** Claudia Marotta, Paola Stefanelli, Fabio Tramuto, Ulrico Angeloni, Carmelo Massimo Maida, Achille Cernigliaro, Teresa Barone, Francesco Vitale, Giovanni Rezza, Walter Mazzucco, For The Sami-Surv Collaboration

**Affiliations:** 1 General Directorate of Health Prevention, Ministry of Health, Rome, Italy; 2 Department of Infectious Diseases, National Institute of Health (ISS), Italy; 3 Department of Health Promotion, Mother and Child Care, Internal Medicine and Medical Specialties “G. D’Alessandro”, University of Palermo, Italy; 4 Clinical Epidemiology Unit and Regional Reference Laboratory of Western Sicily for the Emergence of COVID-19, University Hospital “P. Giaccone”, Palermo, Italy; 5 Regional Health Authority of Sicily, Palermo, Italy; 6 Department of Laboratory Diagnostics, Local Health Unit of Palermo, Palermo, Italy; 7 Division of Biostatistics & Epidemiology Research, Cincinnati Children’s Hospital Medical Center, United States; 8 Davide Alba, Emanuele Amodio, Alessandra Casuccio, Claudio Costantino, Santo Fruscione, Vincenzo Restivo, Alessandra Savatteri (University of Palermo); 9 Nadia D’Agostino, Daniele La Milia, Laura Pecoraro, Claudio Pulvirenti, Domenico Stabile (USMAF Sicilian Unit); 10 Carlo Cesari, Salvatore Zichichi (Italian Red Cross); 11 Alessandra Lo Presti (Italian National Health Institute); 12 Daniela Di Naro, Giorgio Graziano, Giulia Randazzo (COVID-19 western Sicily reference lab); 13 Salvatore Scondotto (Regional Health Authority of Sicily); 14 Stefano Reale, Silvia Scibetta, Fabrizio Vitale (Experimental Zoo-prophylactic Institute of Sicily); 15 Rosario Asciutto, Ranieri Candura, Mariano Lucchese, Giulia Mangano, Maristella Messina, Giuseppa Mistretta, Giulia Palmeri, Antonina Patrizia Rizzo, Antonino Sparaco (Local Health Unit of Trapani); 16 Annalisa Agnone, Francesco Cascio, Daniela Laura Di Quarto, Carmelo Migliorisi (Local Health Unit of Palermo); 17 Stefania D’Amato (Italian Ministry of Health); 18 Vittorio Spoto, Mario Zappia (Local Health Unit of Agrigento).

## Abstract

Despite the pandemic, 34,154 migrants, refugees or asylum-seekers landed in Sicily (Italy) in 2020, representing the main point of entry by sea into Europe. The SARS-CoV-2 surveillance program among migrants arriving to Sicily via the Mediterranean Sea, made by the combination of clinical examination and molecular testing, has been integrated by full-genome sequencing strains using the NGS technology from the last week of February. To date, more than one hundred full-genome strains have been sequenced and 8 different lineages have been identified mostly belonging to the lineages B.1.1.7 and B.1.525. As global access to COVID-19 vaccines should be ensured, the need to provide more detailed information to inform policies and to drive the possible re-engineering of vaccines needed to deal with the challenge of new and future variants should be highlighted.

## Viewpoint

Since the first cases of pneumonia were reported in Wuhan, genomic epidemiology is playing a major role in the characterization and comprehension of SARS-CoV-2 pandemic thanks to the sharing of viral sequences. In particular, the study of the genetic diversity and evolutionary dynamics of SARS-CoV-2 contributes to real-time surveillance and to the understanding of antigenic changes and virus transmission patterns [1]. Moreover, if on one side virus genome sequencing can improve control measures, diagnostic capacity, the detection of infection sources and patterns of infection, disease prognosis, and vaccine effectiveness [2]; it can also provide knowledge on the impact of SARS-CoV-2 variants resistant to neutralization by monoclonal and serum-derived polyclonal antibodies [3]. For all of the aforementioned reasons, in the context of a pandemic, where only global responses may be truly successful, robust amounts of molecular knowledge from SARS-CoV-2 viruses circulating around the world are needed, even in low-resource settings where low-to-absent testing capacity and poor reporting systems are in place [4].

In September 2020, a network of laboratories was launched in Africa by the WHO and the African Centres for Disease Control and Prevention to accelerate genomic-based surveillance for COVID-19 response in the continent [5,6,7,8]. Nevertheless, although the number of genome sequences available in the GISAID from Africa is rapidly increasing, an important information gap should be filled and much remains to be done to build a comprehensive and detailed picture [9].

At the same time, since its inception, the COVID-19 pandemic has shown the immense risks of lacking a global health perspective when coping with such health threats. Despite this, one year after the first COVID-19 documented cases, the lesson is yet to be learnt, with responses running at various speeds in both high and low-middle income countries, in terms of diagnostic and surveillance capacities, health workforce, therapeutic options, and, not least, vaccination campaigns.

**Figure 1:**
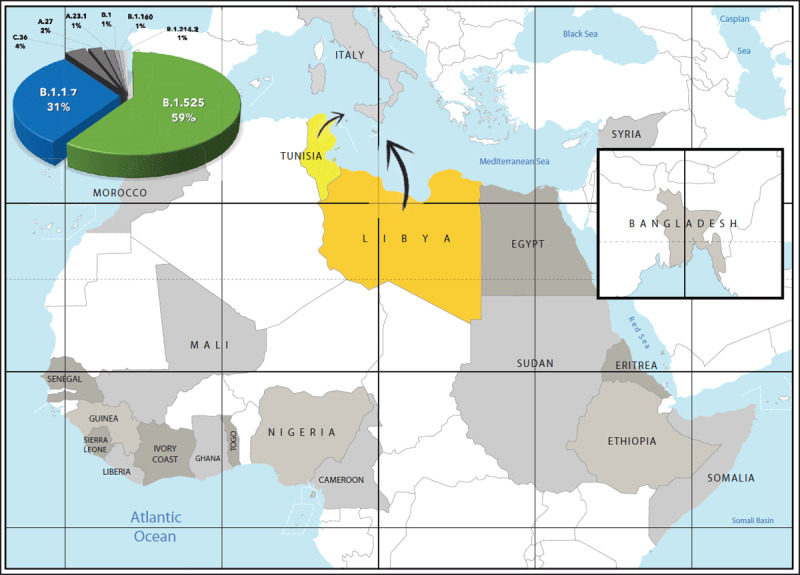
Countries of origin and routes followed by migrants to Europe through the points of entry in Southern Italy.

In this changing scenario, introducing genomic surveillance in migrants arriving to Italy by the Mediterranean Sea via the Tunisian or Libyan routes may have dual/global value. On one hand, it reflects what happens in the place where their journey began or, more often, in the final leg of their very long journey, because for the majority of them Libya represents a last – very long – pause before Europe, bringing migrants from various countries and continents together [10]. On the other hand, through its islands facing the far south (***Figure 1***), Italy is the closest territory to African borders and – counting the 34,154 migrants, refugees or asylum-seekers who arrived in 2020 – represents the main point of entry by sea into Europe [11]. Thus, analysing in depth what happens at this level may improve our understanding of the global SARS-CoV-2 transmission dynamic, particularly in light of the emergence of new variants, while at the same time representing an opportunity to forecast future epidemiological scenarios [12].

To this end, we have scaled up the SARS-CoV-2 surveillance program among migrants arriving to Sicily via the Mediterranean Sea through the Libyan and Tunisian routes, rescued by non-governmental organizations and/or military navy patrolling the area or landed on their own, integrating it with genomics and establishing the SArs-CoV-2 Migrants Surveillance (SAMI-Surv) Collaboration for scientific purposes.

More in depth, since the beginning of pandemic, each single migrant arriving in Italy – already undergoing a medical screening according to the International Health Regulation provisions – has been tested for SARS-CoV-2 with a rapid antigen test at the moment of arrival. If the subject tests positive, a RT-PCR is performed and, if confirmed, the subject is isolated. Regardless of the rapid antigen results, because migrants are all coming from a “high risk area for SARS-CoV-2” according to Italian law, they are all quarantined for 10 days with a second test performed at the end of the period if no symptoms occur before this.

Both isolated and quarantined migrants are hosted in dedicated reception camps or, more often, in reconverted cruise ships organized in areas by cohorts.

Starting from the last week of February 2021, the already ongoing surveillance upon migrants’ arrivals, carried out through the combination of clinical examination and molecular testing as described above, has been integrated with full-genome sequencing strains using the next generation sequencing (NGS) technology. To date, more than one hundred full-genome SARS-CoV-2 sequences have been obtained out of 2,536 positive migrant subjects monitored through the surveillance system in place. Most of them were young males (83.7%, median age 22.5 years old) who followed the Mediterranean Sea routes from low-resource countries (Egypt: 18.5%; Bangladesh: 13.1%; Somalia: 9.8%; Morocco: 7.6%; Tunisia: 6.5%, etc.). Eight different lineages have been identified, mostly belonging to the lineages B.1.525 (59.0%) and B.1.1.7 (31.0%), followed by C.36 (4.0%) and A.27 (2.0%) (***Figure 1***). Interestingly, although the aforementioned lineages have been relatively uncommon in this area of the globe [9], a number of “variants of concern” are beginning to gain more relevance during the most recent period [13]. This body of evidence highlights, on one side, how the phylogenetic classification system is very dynamic in relation to the natural evolutionary and adaptive processes, favouring the emergence of new viral lineages with higher fitness, and, on the other side, the role that genomic surveillance program on migrants arriving from under-sampled regions across the globe can play at their destination countries.

As the SAMI-Surv collaboration also aims to provide further evidence in support of global access to COVID-19 vaccines [14], more detailed information from our experience will be shared soon with the hope of providing early strategic information to national and global scientific communities to inform policies and, potentially, to drive the re-engineering of vaccines needed to deal with the challenge of new and future variants [15].
